# EEG monitoring during anesthesia in children aged 0 to 18 months: amplitude-integrated EEG and age effects

**DOI:** 10.1186/s12887-022-03180-x

**Published:** 2022-03-26

**Authors:** Barbara Schultz, Michael Schultz, Martin Boehne, Nils Dennhardt

**Affiliations:** 1 Department of Anesthesiology and Intensive Care Medicine, Hannover Medical School, Hannover, Germany; 2 Medical University of Vienna, Vienna, Austria; 3 Department of Pediatric Cardiology and Intensive Care Medicine, Hannover Medical School, Hannover, Germany

**Keywords:** aEEG, Sedation, Anesthesia, Classification, Electroencephalogram, Pediatric ICU

## Abstract

**Background:**

The amplitude-integrated EEG (aEEG) is a widely used monitoring tool in neonatology / pediatric intensive care. It takes into account the amplitudes, but not the frequency composition, of the EEG. Advantages of the aEEG are clear criteria for interpretation and time compression. During the first year of life, the electroencephalogram (EEG) during sedation / anesthesia changes from a low-differentiated to a differentiated EEG; higher-frequency waves develop increasingly. There are few studies on the use of aEEG during pediatric anesthesia. A systematic evaluation of the aEEG in defined EEG stages during anesthesia / sedation is not yet available. Parameters of pediatric EEGs (power, median frequency, spectral edge frequency) recorded during anesthesia and of the corresponding aEEGs (upper and lower value of the aEEG trace) should be examined for age-related changes. Furthermore, it should be examined whether the aEEG can distinguish EEG stages of sedation / anesthesia in differentiated EEGs.

**Methods:**

In a secondary analysis of a prospective observational study EEGs and aEEGs (1-channel recordings, electrode positions on forehead) of 50 children (age: 0–18 months) were evaluated. EEG stages: A (awake), Slow EEG, E_2_, F_0_, and F_1_ in low-differentiated EEGs and A (awake), B_0–2_, C_0–2_, D_0–2_, E_0–2_, F_0–1_ in differentiated EEGs.

**Results:**

Median and spectral edge frequency increased significantly with age (*p* < 0.001 each). In low-differentiated EEGs, the power of the Slow EEG increased significantly with age (*p* < 0.001). In differentiated EEGs, the power increased significantly with age in each of the EEG stages B_1_ to E_1_ (*p* = 0.04, or less), and the upper and lower values of the aEEG trace increased with age (*p* < 0.001). A discriminant analysis using the upper and lower values of the aEEG showed that EEG epochs from the stages B_1_ to E_1_ were assigned to the original EEG stage in only 19.3% of the cases. When age was added as the third variable, the rate of correct reclassifications was 28.5%.

**Conclusions:**

The aEEG was not suitable for distinguishing EEG stages above the burst suppression range. For this purpose, the frequency composition of the EEG should be taken into account.

## Introduction

The amplitude-integrated EEG (aEEG) is a method for the time-compressed display of electroencephalographic (EEG) recordings [1]. It contains information about EEG amplitudes, but not about the frequency composition of the EEG. The aEEG is an established monitoring method in neonatology [1]. It is used for assessment of seizures and background cerebral activity in high-risk neonates [2]. The background activity can be interpreted by means of 5 different patterns [1]. Typically, an aEEG recording comprises 1 or 2 EEG channels [3]. Advantages of the aEEG are the clear criteria for interpretation and the time compression. Compared to conventional multichannel EEG recordings, establishing and maintaining such a recording requires less effort. In addition to being used for newborns, aEEG devices are also used in pediatric intensive care patients beyond the neonatal age [4]. In a recent survey, main indications were neurological complications or disease and altered mental state [4].

The background activity of the EEG can be changed in pediatric patients due to illness. Another factor that affects background activity is sedation. Increasing doses of sedative substances, administered intravenously or by inhalation, lead to typical changes in the EEG. These changes, which, e.g., occur with propofol and with inhalation anesthetics such as sevoflurane, consist in a progressive slowing up to the occurrence of intermittent suppression phases and finally to a continuous suppression of the EEG [5, 6]. The EEG changes can be described using a stage classification, and they can be used to assess the depth of sedation and anesthesia [7, 8].

Changes in the EEG caused by brain maturation must be taken into account when assessing sedation effects in the child’s EEG. An age effect that can be observed in the pediatric EEG during anesthesia is an increase in the power of the EEG with increasing age. This effect was described for example in a study in children aged 0 to 17 months [9] and in another study comparing two groups of children aged 0–3 months and 4–6 months, respectively [10]. During the first few months of life, the frequency composition of the EEG during anesthesia changes. While negligible frontal alpha power (8–12 Hz) was found in children aged 0–3 months, increased alpha power was present in children aged 4–6 months [10]. Furthermore, it was reported that theta (4–8 Hz) and alpha (8–12 Hz) oscillations emerge by ~ 4 months, that alpha oscillations increased in power from 4 to 10 months, and that frontal alpha-oscillation predominance emerged at ~ 6 months [11].

In a study by de Heer et al. [12], correlations between end-tidal sevoflurane concentrations and the relative power in EEG frequency bands were not found in children up to 6 months, while such correlations were observed in children from 6 to 12 months and 1–6 years. In another study, obvious concentration-dependent changes in EEG parameters, including the spectral edge frequency, were observed in children aged 6 months to 2 years during sevoflurane anesthesia, while there were few changes in children younger than 3 months [13]. Several studies have examined EEG changes during emergence from anesthesia [14–16]. In some of the studies, little or no changes in power [14, 15] were found in younger children, while changes were seen in older children, with the upper age limit of the younger children in these studies being 3 or 6 months. On the other hand, Cornelissen et al. [16] described that, in children with an age of less than 3 months, frontal EEG frequency bands shifted in power with decreasing end-tidal sevoflurane.

There are few studies on the use of aEEG during anesthesia in children [14, 15, 17–19]. A systematic evaluation of the aEEG with regard to defined EEG stages during anesthesia is not yet available.

In most children, at the age of 6 months, the EEG is so far developed that during sedation and anesthesia a range of EEG stages, that is analogous to EEG stages of older children and adults, can be distinguished [20]. The terms low-differentiated EEG and differentiated EEG were introduced to describe if in an EEG, due to its developmental state, the full range of these EEG stages cannot or can be distinguished [20, 21].

The aEEG is used in many pediatric intensive care units [4]. For its use in children with differentiated EEGs knowledge on how sedative drugs affect the aEEG is important. In addition to its use in the intensive care unit, the aEEG might be a valuable addition to the monitoring of pediatric patients in the operating room.

In this analysis, EEGs recorded during administration of sevoflurane in children between the ages of 0 and 18 months were examined in order to gain information about age-related changes in the EEG and the aEEG. Furthermore, it was assessed whether aEEG in children with differentiated EEG is suitable to distinguish between different EEG stages of sedation and anesthesia.

## Patients and methods

### Data basis

As data basis for the analysis, EEG and aEEG recordings from a previous, single-center, prospective, observational trial published by Dennhardt et al. were used [21]. The study had been approved by the responsible ethics committee (Ethics Committee of Hannover Medical School, Germany, Chairperson Prof. Dr. H. D. Troeger, No. 3259–2016 dated June 22, 2016). Because of the observational design without change of the anesthetic management, written consent was not necessary according to the Ethics Committee. Children between 0 and 24 months old undergoing elective surgery were eligible for the study. Excluded were children who had a neurological disorder or a severe developmental delay, or who were preoperatively given medication that altered the EEG, with the exception of midazolam as premedication. The preoperative physical status was scored according to the American Society of Anesthesiologists (ASA) physical status scale. Sevoflurane and remifentanil were administered during the steady state of anesthesia after induction with sevoflurane, remifentanil and atracurium. Anesthetic management followed the usual practice.

### aEEG

**Fig. 1:**
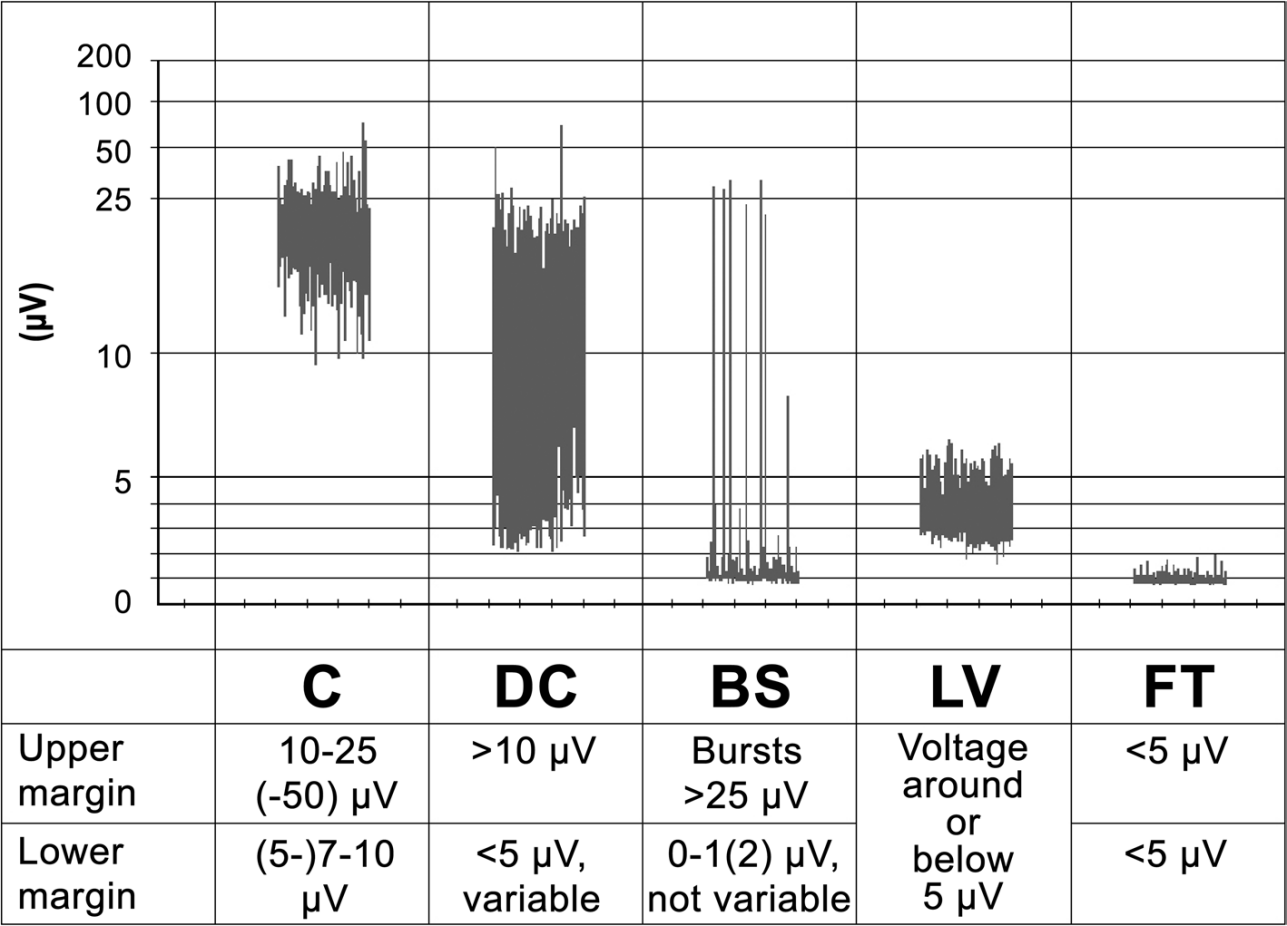
aEEG background patterns (C: Continuous, DC: Discontinuous, BS: Burst Suppression, LV: Low Voltage, FT: Flat Trace). Definitions according to Hellström-Westas et al. [22]. According to Schettler, for LV the lower margin is around or below 5 μV and the upper margin is no more than 10 μV [3]. The assignment of an aEEG section to an aEEG pattern is based on the upper and lower margins of the aEEG. The visual determination of the position of the upper or lower margin for an aEEG section can be carried out in such a way that 50% of the associated values are above and 50% below [26]

The aEEG contains information about the amplitudes of an EEG [1]. The calculation steps of the aEEG include filtering, rectification, smoothing, and time compression of the EEG. Over time, a band-shaped pattern results, the upper margin of which represents the highest amplitudes and the lower margin of which represents the lowest amplitudes of successive EEG epochs. The aEEG is represented using a so-called semilogarithmic scale, i.e., the scale is linear up to 10 μV, and logarithmic above [1, 22–24]. For the use of the aEEG in neonatology, there are suggestions for distinguishing different patterns based on the position of the upper and lower margin of the aEEG trace [25]. There are 5 patterns in the classification by Hellström-Westas et al. [22]. Figure 1 shows examples of these patterns from the own data set.

### EEG recording, stage classification, spectral parameters, and aEEG parameters

**Fig. 2:**
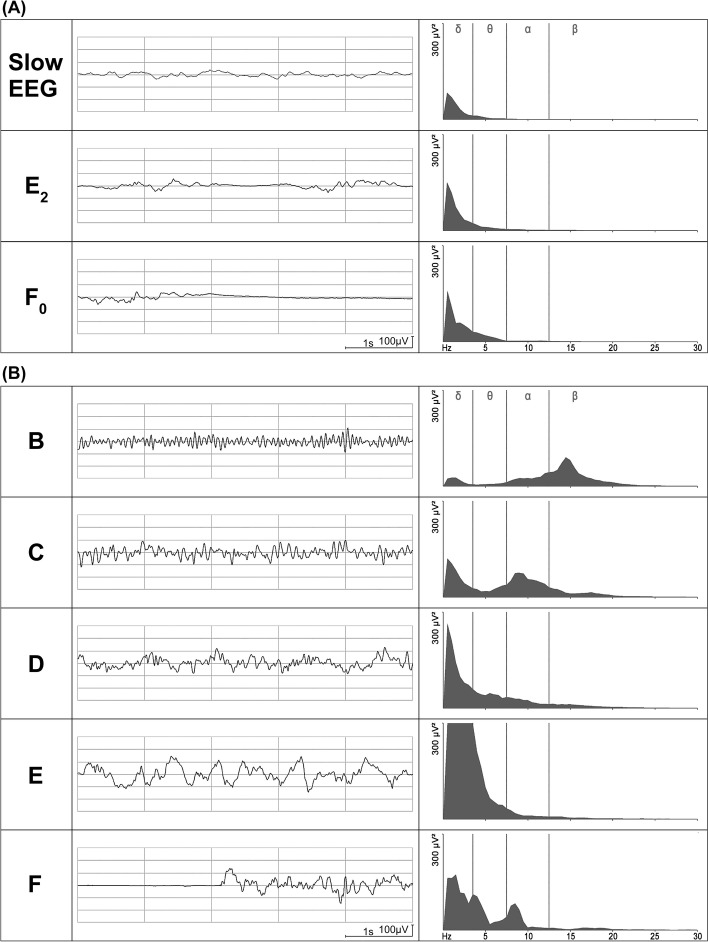
Raw EEGs and power spectra recorded during anesthesia. The power spectra were calculated for 20s epochs. **A** Low-differentiated EEG, 1-month-old child. Examples of the Slow EEG, E_2_, and F_0_ stages. **B** Differentiated EEG, 11-month-old child. Examples of stages B, C, D, E, and F

The EEGs were recorded using the Narcotrend-Compact M (MT MonitorTechnik, Bad Bramstedt, Germany) as 1-channel registrations, for which the required three electrodes were attached to the patient’s forehead. The Narcotrend-Compact M checks the anesthetic EEG in children in the first year of life to determine whether the EEG is either low-differentiated or differentiated. In the case of low-differentiated EEGs, the stages A (awake), Slow EEG, E_2_, F_0_, and F_1_ are used. The Slow EEG is characterized by continuous activity without suppression periods. Suppression periods begin to occur in stage E_2_, and the length of suppression periods increases in stages E_2_, F_0_, and F_1_. In the case of differentiated EEGs, the Narcotrend-Compact M distinguishes the stages A (awake), B_0–2_, C_0–2_, D_0–2_, E_0–2_, F_0–1_, and it calculates the Narcotrend Index between 100 and 0 [7, 8]. Figure 2A and B show examples of different EEG stages in a low-differentiated and in a differentiated EEG recorded during anesthesia in a 1-month-old and in an 11-month-old child. The associated power spectra show the power of frequency components of the EEG. Regardless of the degree of differentiation, the Narcotrend-Compact M calculates the aEEG, the density spectral array (DSA), and other parameters.

As basis for this evaluation, the original recordings were classified with the Narcotrend software (Version 3.4). 50 EEG recordings with aEEG courses were reanalyzed, 11 recordings were not available due to technical reasons.

The following EEG parameters were considered: the EEG power (μV) (calculated as root of the signal power (μV^2^)), the median frequency (50% quantile of the power spectrum), and the spectral edge frequency (SEF95, 95% quantile of the power spectrum). Furthermore, the two aEEG parameters upper and lower value of the aEEG trace, which are calculated by the Narcotrend-Compact M, were evaluated. All parameters, the Narcotrend stage, and the Narcotrend Index were calculated for consecutive 20 s epochs. For each of the 50 patients, the entire EEG during the steady state (cut to suture) was evaluated. To characterize the courses in the steady state, a mean value was calculated from the 20 s values for each patient and for the parameters median frequency, SEF95, power, and Narcotrend Index.

Tinning and Acworth (2007) developed formulas for calculating the mean body weight in children of different ages [27]. The children’s documented body weight and the calculated weight according to Tinning and Acworth [27] were used in analyses.

### Statistics

The calculations were carried out with the statistical software SAS (version 9.3). Fisher’s exact test was used to compare nominal data. The t-test was used for mean value comparisons for two groups. Linear regression models were used to investigate the relationship between age and the parameters EEG power, and the upper and the lower value of the aEEG trace. Spearman’s rank correlation was used to examine correlations between EEG spectral parameters (median and SEF95), patients’ age and patients’ weight.

A multiple correlation coefficient between the Narcotrend Index and the upper and the lower values of the aEEG trace was calculated.

By way of a linear discriminant analysis, it was evaluated whether the aEEG parameters upper and lower value of the aEEG trace are suitable for distinguishing the stages B_1_ to E_1_ of the differentiated EEG; an additional linear discriminant analysis was carried out with the patient’s age as third parameter. The sample size was *n* = 10,153.

As part of the discriminant analyses, reclassifications into the stages B_1_ to E_1_ were carried out.

For the discriminant analyses of the differentiated EEGs, stage B_0_ was not considered due to the small number of cases.

The awake EEG was not included in the analyses; furthermore, EEGs characterized by intermittent or continuous suppression periods were not included as for such EEGs defined patterns exist in the aEEG classification by Hellström-Westas et al. [22].

The significance threshold was assumed to be *p* < 0.05.

## Results

Of the 50 patients, 21 had a low-differentiated EEG according to the Narcotrend evaluation and 29 patients had a differentiated EEG.

### Demographic data

The included 50 patients and the not included 11 patients did not differ significantly with regard to age (*p* = 0.96), documented weight (*p* = 0.96), and sex (*p* = 1.00). There was no evidence that the 11 patients not included in the analysis could skew the results of the study.

The patients with low-differentiated EEGs were younger and lighter than the children with differentiated EEGs (age: 2.5 ± 1.2 vs. 9.7 ± 4.3 months, weight: 4.9 ± 1.2 vs. 8.5 ± 2.0 kg; each *p* < 0.001). All patients were under 19 months of age. Regarding the ASA status (ASA: American Society of Anesthesiologists), i.e., the preoperative physical condition, there was no difference between the two patient groups (*p* = 0.84).

The documented weight was significantly lower than the calculated weight according to Tinning and Acworth [27], both in children with a low-differentiated EEG (*p* = 0.007) and children with a differentiated EEG (*p* = 0.04). The difference between documented and calculated weight was 11.9% (mean ± standard deviation: 0.6 ± 0.9 kg) in the group with low-differentiated EEGs and 8.4% (mean ± standard deviation: 0.7 ± 1.7 kg) in the group with differentiated EEGs.

### EEG spectral parameters, patients’ age, and patients’ weight

**Fig. 3:**
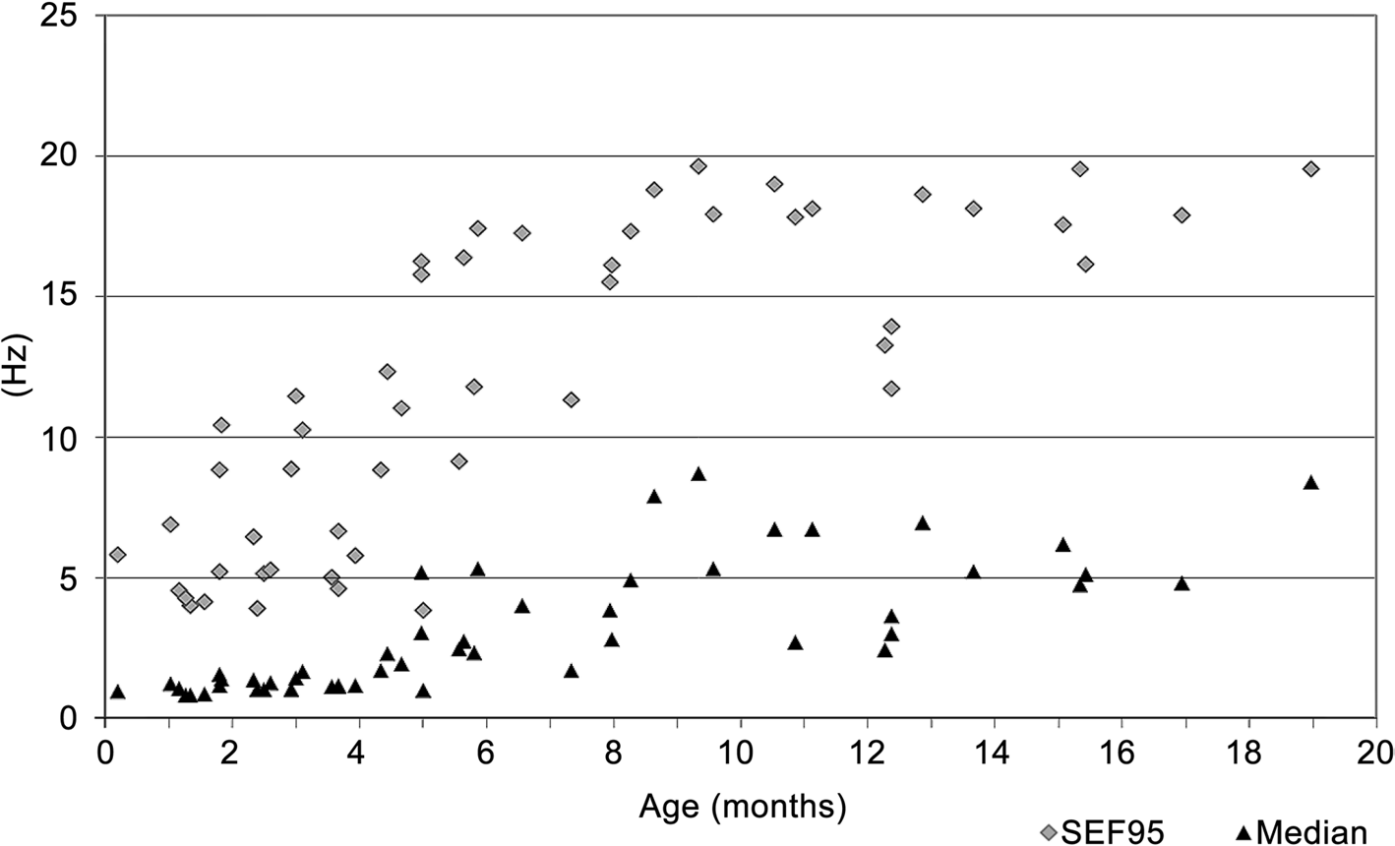
Change in spectral edge frequency (SEF95) and median frequency with age

For the course of each anesthesia, the means of median frequency and spectral edge frequency (SEF95) were calculated as parameters from the frequency range. The median frequency and SEF95 increased significantly with age (*p* < 0.001 each) (Fig. 3). The median frequency and the spectral edge frequency also increased significantly with the patients’ documented body weight and with the calculated weight according to Tinning and Acworth [27] (*p* < 0.001 each).

**Fig. 4:**
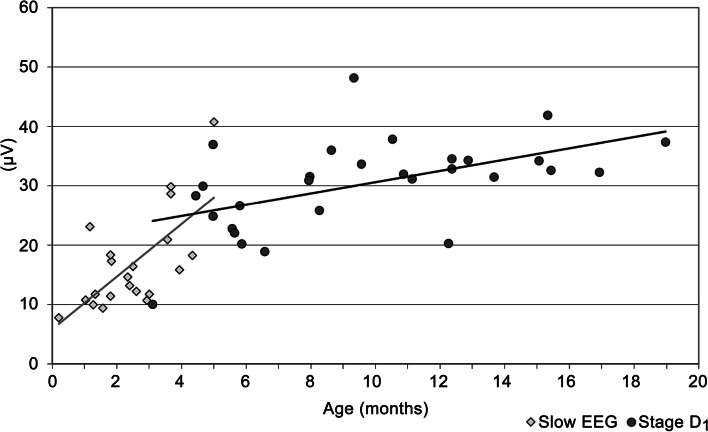
EEG power, dependent on age. Slow EEG and Stage D_1_

In children with low-differentiated EEGs, 89.9% of the classifications for the steady state of anesthesia were Slow EEG, 7.5% were E_2_, 2.6% were F_0_, and 0.1% were F_1_. There was a significant increase in power of the Slow EEG with age (*p* < 0.001) (Fig. 4).

For each of the differentiated EEGs, the median Narcotrend Index was calculated as a measure to describe the mean depth of hypnosis at steady-state anesthesia. The mean of all 29 medians was 53.6 (IQR = (48.5; 62)), which can be assigned to stage D_1_.

For the children with differentiated EEGs, a relation between age and EEG power was examined for the stages B_1_ to E_1_. First, the mean EEG power was calculated for each child and for each stage. In each of the stages, the power increased significantly with age (B_1_: *p* = 0.04, B_2_: *p* = 0.004, C_0_: *p* = 0.001, C_1_: *p* < 0.001, C_2_: *p* < 0.001, D_0_: *p* = 0.04, D_1_: *p* = 0.003, D_2_: *p* = 0.007, E_0_: *p* = 0.004, E_1_: *p* = 0.005). Figure 4 shows, as an example, the increase in power with age for stage D_1_.

### aEEG parameters and patients’ age

Accompanying the described increase in power in the stages, the upper and lower values of the aEEG increased significantly with age in each of the stages B_1_ to E_1_ (in each of the stages B_1_ to E_1_ upper values *p* < 0.001, lower values *p* < 0.001).

### aEEG parameters and EEG stages

**Fig. 5:**
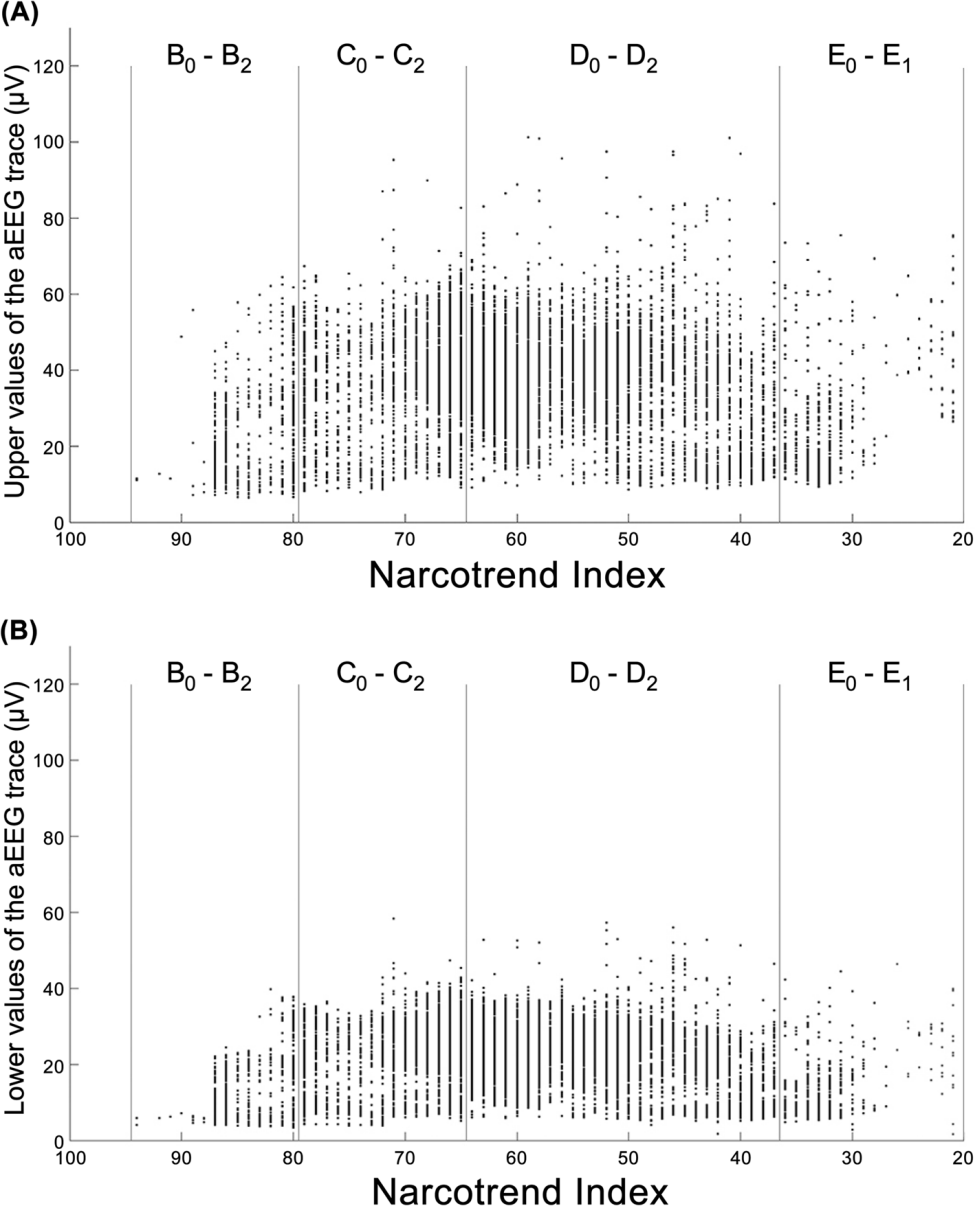
Values of the aEEG in the stages B_0_ to E_1_. **A** Upper values of the aEEG trace. **B** Lower values of the aEEG trace

**Fig. 6:**
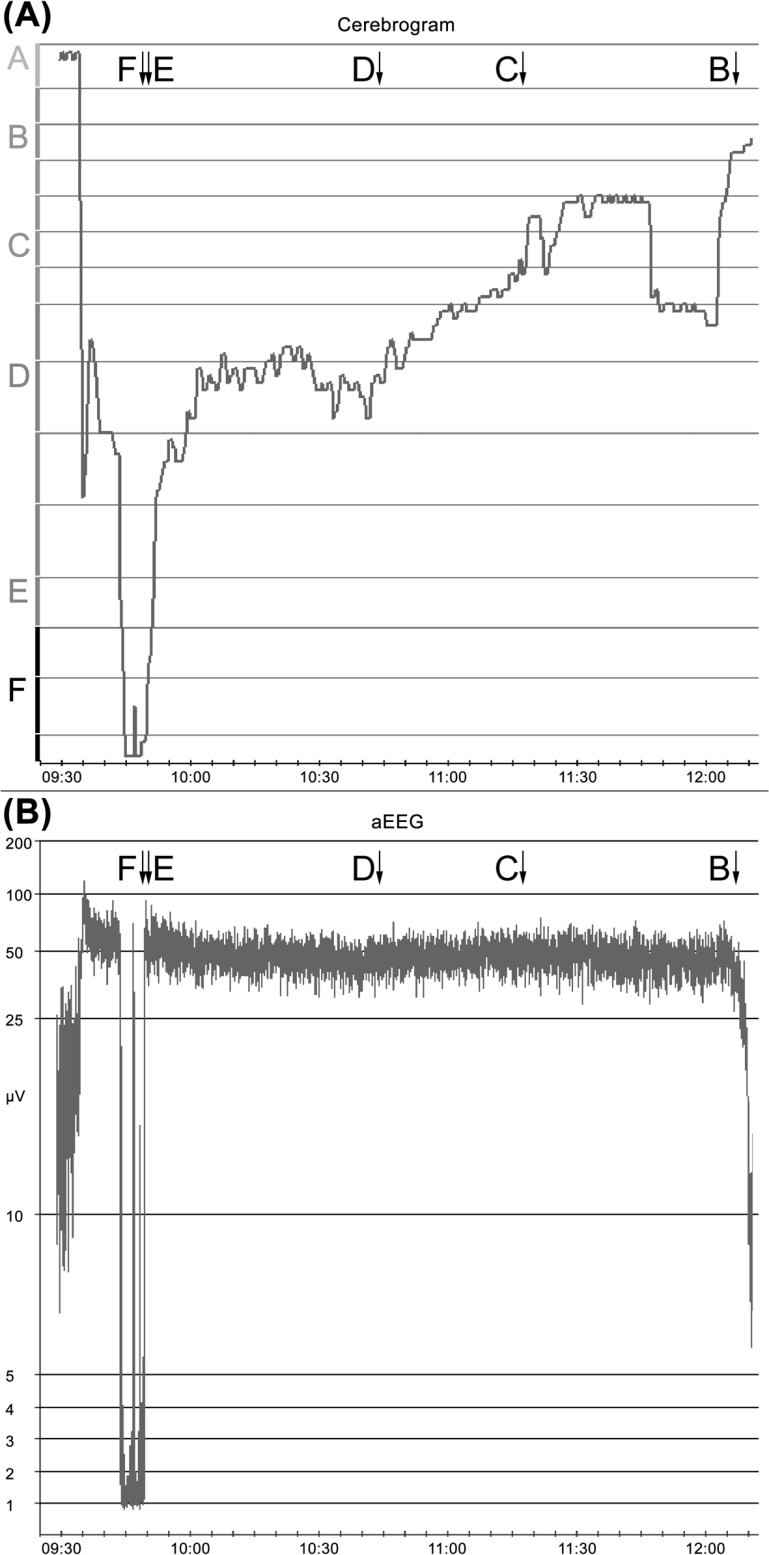
Course of anesthesia in an 11-month-old child. **A** EEG stages and index. **B** aEEG. The shift to lower μV values that occurred at the end of the aEEG course is due to very low EEG amplitudes in stage B_1_. The raw EEGs shown in Fig. 2B originate from this course of anesthesia. The markings show the times at which the raw EEG sections were drawn

In order to analyze whether there is a correlation between EEG stages B_1_ to E_1_ and the aEEG in the differentiated EEGs, a multiple correlation coefficient between the Narcotrend Index and the upper and the lower values of the aEEG trace was calculated. The correlation coefficient was r = 0.09. In Fig. 5A and B, no relationship with the Narcotrend Index can be seen. As an example, Fig. 6 shows the course of the EEG stages or EEG index values (cerebrogram) and the aEEG from one course of anesthesia in an 11 month-old child. EEG stages from the E to the B range occurred between 09:50 and 12:00 h. In spite of the different EEG stages, there are no significant changes in the aEEG during this period. (The raw EEG segments shown in Fig. 2B were taken from the course of anesthesia shown in Fig. 6 at the marked times.)

By way of a linear discriminant analysis, it was evaluated whether the upper and the lower values of the aEEG trace are suitable for distinguishing the EEG stages B_1_ to E_1_. For each of the EEG stages from B_1_ to E_1_, it was checked which of these stages an EEG epoch was, based on the two associated aEEG parameters, assigned to. A correct classification was made in 19.3% of the cases. In 80.7% of the cases, an incorrect stage was chosen. When age was added as the third variable, the rate of correct reclassifications was 28.5%.

Table 1A and B show the results (as percentages) of the classification into the substages B_1_-E_1_ by means of discriminant analysis without (Table 1A) and with (Table 1B) patient age as additional variable. Correct classifications are indicated by boldface. The total number of epochs per substage is indicated in an extra column on the left side of each table. The total number of all epochs is 10,153.

**Table 1:** Results of the classification based on A) upper and lower value of the aEEG, B) upper and lower value of the aEEG plus patient age

**(A)**			**Classification based on aEEG parameters**									
	**n**		**B**_1_	**B**_**2**_	**C**_**0**_	**C**_**1**_	**C**_**2**_	**D**_**0**_	**D**_**1**_	**D**_**2**_	**E**_**0**_	**E**_**1**_
**Classification according to Narcotrend-Compact M**	332	**B**_**1**_	**66%**	0%	0%	0%	0%	15%	1%	0%	11%	6%
	322	**B**_**2**_	34%	**0%**	0%	0%	11%	26%	4%	0%	18%	7%
	447	**C**_**0**_	31%	0%	**0%**	0%	17%	28%	9%	1%	7%	8%
	489	**C**_**1**_	22%	0%	0%	**0%**	18%	30%	13%	0%	8%	9%
	1452	**C**_**2**_	13%	0%	0%	0%	**27%**	35%	15%	0%	3%	7%
	3040	**D**_**0**_	17%	0%	0%	0%	14%	**42%**	17%	0%	5%	6%
	2285	**D**_**1**_	11%	0%	0%	0%	19%	35%	**24%**	0%	4%	7%
	1265	**D**_**2**_	41%	0%	0%	0%	5%	23%	10%	**1%**	7%	13%
	479	**E**_**0**_	74%	0%	0%	0%	1%	8%	2%	0%	**7%**	9%
	42	**E**_**1**_	31%	0%	0%	0%	14%	10%	10%	0%	10%	**26%**
**(B)**			**Classification based on aEEG parameters and age**									
	**n**		**B**_1_	**B**_**2**_	**C**_**0**_	**C**_**1**_	**C**_**2**_	**D**_**0**_	**D**_**1**_	**D**_**2**_	**E**_**0**_	**E**_**1**_
**Classification according to Narcotrend-Compact M**	332	**B**_**1**_	**71%**	6%	1%	2%	0%	9%	0%	0%	6%	5%
	322	**B**_**2**_	30%	**21%**	1%	1%	4%	12%	3%	0%	19%	8%
	447	**C**_**0**_	8%	14%	**5%**	2%	15%	13%	6%	2%	28%	7%
	489	**C**_**1**_	9%	4%	3%	**3%**	17%	27%	9%	3%	19%	5%
	1452	**C**_**2**_	2%	4%	4%	3%	**25%**	30%	12%	4%	14%	4%
	3040	**D**_**0**_	3%	6%	2%	1%	11%	**44%**	9%	5%	16%	3%
	2285	**D**_**1**_	2%	4%	6%	1%	20%	31%	**14%**	6%	11%	3%
	1265	**D**_**2**_	5%	2%	3%	2%	6%	20%	3%	**14%**	39%	5%
	479	**E**_**0**_	9%	2%	6%	0%	2%	0%	0%	6%	**69%**	6%
	42	**E**_**1**_	7%	7%	5%	2%	12%	2%	5%	17%	24%	**19%**

## Discussion

The current analysis focused on age-related changes in the EEG and the aEEG and on the ability of aEEG-derived parameters to distinguish between different EEG stages of sedation and anesthesia. It was shown for the first time that the increase in power with age occurs across all EEG sub-stages from very light anesthesia / sedation (B_1_) to very deep anesthesia / sedation (E_1_). In the differentiated EEGs, EEG stages from the range B_1_ to E_1_ could neither be distinguished on the basis of the upper and lower values of the aEEG trace, nor on the basis of the two aEEG parameters and the patient’s age. This result supports the view that, in EEG stages above the burst suppression range, the aEEG is of limited value for depth of sedation and anesthesia monitoring in children aged 0–18 months.

Growth and maturation of the brain are particularly rapid in the first year of life [28]. The observed increase in median frequency and SEF95 in both low-differentiated and differentiated EEGs can be explained on the one hand by the development of higher frequencies in the EEG with advancing age. On the other hand, the median frequency and SEF95 depend on the intraoperative anesthetic EEG stage. The increase in power with age is consistent with observations made by other authors [9, 10, 15]. Sankupellay et al., who observed a rise of spectral power in different stages of sleep within the first two years of life, speculated that these changes of the spectral power represented a sleep state-independent aspect of EEG development and were related to structural brain development [29].

In the present analysis, based on the upper and lower values of the aEEG trace, EEG stages in the range from stage B_1_ to E_1_ were not reliably distinguished in children with differentiated EEGs, even when additionally considering the children’s age. When calculating the aEEG, one of the calculation steps is filtering the EEG. This is done in order to reduce the effect of artifacts [30]. An asymmetrical bandpass filter is used, which strongly suppresses activity below 2 Hz and above 15 Hz [22–24]. The primary feature of the B stages of the anesthetic EEG are waves in the beta frequency range, i.e., above 12.5 Hz. The anesthetic EEG stages from D_0_ to E_1_ are characterized by increasingly pronounced delta activity (0.5–3.5 Hz). Due to the filtering, part of the waves from the frequency ranges, which are characteristic for the B range and for the D / E range, are therefore not represented in the aEEG. This could have contributed to the fact that in our investigation it was not possible to distinguish between stages in the B_1_ to E_1_ range using the aEEG. The frequency composition of the EEG, which is the essential characteristic for distinguishing the anesthetic EEG stages, is not taken into account in the aEEG. Figure 2B illustrates that the raw EEGs differ significantly in their frequency composition. A distinction between stages C and D based on the amplitudes is not possible. However, in the examples in Fig. 2B, there are higher amplitudes in stage E, while in stage B there is a tendency towards lower amplitudes. In B stages, the EEG amplitude can be very small, so that the upper and lower values of the aEEG trace can consequently assume small values (see also Fig. 5A and B). Such portions of the aEEG originating from EEGs with high-frequency waves and low amplitude may fulfil the criteria of the aEEG pattern Low Voltage (LV). A Low Voltage pattern in the aEEG is associated with an unfavorable prognosis after perinatal asphyxia [31, 32] and is classified as severely abnormal [32]. A Low Voltage pattern in the B stage of the anesthetic EEG is a normal finding.

Figure 5A shows that the range of values for the upper values of the aEEG is similar across stages B_1_ to E_1_, with a tendency towards smaller values in the B and E stages. The same applies to the lower values of the aEEG (Fig. 5B). Figure 5A and B show that the upper and the lower values of the aEEG provide little EEG stage-specific information. The reclassification results obtained by means of the linear discriminant analysis (Table 1A and B) confirm this visual assessment. Apart from linear discriminant analysis, other methods, e.g., neuronal networks, are available for classification tasks [33]. However, such alternative methods would also be limited by the information content of the available variables.

Although the aEEG is a widely used monitoring tool in neonatology and pediatric intensive care [4], so far use of the aEEG for sedation monitoring was only reported by some authors [34, 35]. Reports on the use of the aEEG during anesthesia differ in their estimation of the value of the aEEG for depth of anesthesia monitoring [17–19]. In children in the first two years of life, absorption, distribution, and excretion of medication change due to maturation. There are also pharmacodynamic maturation processes [36]. This makes individually adequate dosing difficult. Even though aEEG is not suitable for reliably distinguishing between stages in the B_1_ to E_1_ range, it can support the detection of burst suppression EEGs as well as complete EEG suppression (stages F_0_ and F_1_). For these EEG patterns, which can occur at higher doses of hypnotically acting substances, the classes according to Hellström-Westas et al. [22] include the classes Burst Suppression (BS) and Flat Trace (FT). In a guideline on postoperative delirium, the European Society of Anaesthesiology recommends avoiding burst suppression patterns in adults during anesthesia [37]. A consensus statement on the role of neuromonitoring on perioperative outcomes also recommends avoiding burst suppression EEGs [38]. It should, however, be borne in mind that, in preterm infants, an EEG pattern with intermittent, very flat sections can be a normal finding due to development. A pattern with alternating phases of higher amplitude and flatter amplitude (tracé alternant) can still be observed in healthy deep sleep in cerebrally healthy children up to 44–46 weeks of postconceptional age [39].

In neonatology, the aEEG is a standard procedure for patient monitoring [1]. The aEEG is also used for intensive medical care for preterm infants [1, 40] and for patients beyond the age of newborns [41]. When sedating as part of neonatal intensive care, the aEEG can help to identify and avoid very deep stages of sedation [35]. If the aEEG is used in older children in the pediatric intensive care unit or in the operating room, then the relationships described in this work should be taken into account. If one wants to use the aEEG for older children in the context of sedation or anesthesia, then the inclusion of information about the proportion of higher frequency waves in the EEG helps to distinguish between EEG stages that fall within the range of the Continuous pattern according to Hellström-Westas et al. [22]. In the case of the Low Voltage pattern according to Hellström-Westas et al. [22], the content of higher-frequency waves can be used to decide whether the EEG stage is one of very light or very deep sedation or anesthesia.

A limitation of the analysis is the fact that a power calculation was not provided as the analysis was performed on a pre-existing data base.

Interesting future research topics arise with regard to the clinical application of the aEEG and to possible methodological modifications and improvements of the aEEG:

The criteria by Hellström-Westas et al. for aEEG classification were developed for preterm and term neonates [22]. Other classifications have also been proposed for this age group [4, 22], while no classification of the aEEG was developed specifically for older children [4]. In recent literature, there have been demands to identify normal amplitudes of the aEEG in children of different ages [42, 43] and to analyze how sedation affects the aEEG [42].

When the aEEG is calculated, the EEG signal is filtered, rectified, smoothed, and time-compressed. It has been suggested to extract amplitude values from the non-rectified EEG signal, to use less strict filters, and to change the semilogarithmic scale of the aEEG [44].

Methods that provide information about the frequency content of the EEG should be included in aEEG monitoring, such as the density spectral array.

## Conclusions

In summary, it can be stated that the age-related increase of the EEG power is accompanied by changes of the upper and lower values of the aEEG. We performed a systematic evaluation of the aEEG with regard to defined EEG stages of anesthesia. Such an evaluation has not been made so far. The analyses carried out show that, even when considering the patient’s age, the aEEG cannot be used to distinguish between differentiated EEG stages that lie between the awake EEG and the EEG with emerging suppression periods. This does not diminish the value of the aEEG when used for other indications. For a discrimination of EEG stages of depth of anesthesia / sedation above the burst suppression range, the frequency composition of the EEG should be taken into account. It remains to be investigated whether modifications to the calculation of the aEEG improve the information content of the aEEG.

## Data Availability

The datasets used and/or analysed during the current study are available from the corresponding author on reasonable request.

## Abbreviations

aEEG: Amplitude-Integrated Electroencephalogram
ASA: American Society of Anesthesiologists
BS: Burst Suppression
C: Continuous
DC: Discontinuous
EEG: Electroencephalogram
FT: Flat Trace
IQR: Interquartile Range
LV: Low Voltage
SEF95: Spectral Edge Frequency 95%
SAS: Statistical Analysis System
